# Antibacterial-Integrated Collagen Wound Dressing for Diabetes-Related Foot Ulcers: An Evidence-Based Review of Clinical Studies

**DOI:** 10.3390/polym12092168

**Published:** 2020-09-22

**Authors:** Ibrahim N. Amirrah, Mohd Farhanulhakim Mohd Razip Wee, Yasuhiko Tabata, Ruszymah Bt Hj Idrus, Abid Nordin, Mh Busra Fauzi

**Affiliations:** 1 Centre for Tissue Engineering and Regenerative Medicine, UKM Medical Centre, Jalan Yaacob Latiff, Bandar Tun Razak, Cheras, Kuala Lumpur 56000, Malaysia; noramirrahibrahim@gmail.com (I.N.A.); ruszyidrus@gmail.com (R.B.H.I.); 2 Institute of Microengineering and Nanoelectronics, Universiti Kebangsaan Malaysia, Bangi 43600, Selangor, Malaysia; m.farhanulhakim@ukm.edu.my; 3 Laboratory of Biomaterials, Department of Regeneration Science and Engineering, Institute for Frontier Life and Medical Sciences, Kyoto University, Kyoto 606-8397, Japan; yasuhiko@infront.kyoto-u.ac.jp; 4 Department of Physiology, Faculty of Medicine, Universiti Kebangsaan Malaysia, Cheras, Kuala Lumpur 56000, Malaysia; m.abid.nordin@gmail.com

**Keywords:** antibacterial, diabetic ulcer, collagen, wound dressing, wound healing, clinical trial

## Abstract

Diabetic foot ulcer (DFU) is a chronic wound frequently delayed from severe infection. Wound dressing provides an essential barrier between the ulcer and the external environment. This review aimed to analyse the effectiveness of antibacterial collagen-based dressing for DFU treatment in a clinical setting. An electronic search in four databases, namely, Scopus, PubMed, Ovid MEDLINE(R), and ISI Web of Science, was performed to obtain relevant articles published within the last ten years. The published studies were included if they reported evidence of (1) collagen-based antibacterial dressing or (2) wound healing for diabetic ulcers, and (3) were written in English. Both randomised and non-randomised clinical trials were included. The search for relevant clinical studies (*n*) identified eight related references discussing the effectiveness of collagen-based antibacterial wound dressings for DFU comprising collagen impregnated with polyhexamethylene biguanide (*n* = 2), gentamicin (*n* = 3), combined-cellulose and silver (*n* = 1), gentian violet/methylene blue mixed (*n* = 1), and silver (*n* = 1). The clinical data were limited by small sample sizes and multiple aetiologies of chronic wounds. The evidence was not robust enough for a conclusive statement, although most of the studies reported positive outcomes for the use of collagen dressings loaded with antibacterial properties for DFU wound healing. This study emphasises the importance of having standardised clinical trials, larger sample sizes, and accurate reporting for reliable statistical evidence confirming DFU treatment efficiency.

## 1. Introduction

Diabetes mellitus (DM) presents a large social, financial, and health system burden globally; it is estimated to affect 700 million people by 2045 [1,2]. The morbidity and mortality in the diabetic population are mainly caused by the complications raised from the severe hyperglycaemia. A common complication of DM is the slow or non-healing of wounds, particularly in the lower extremity. It has been estimated that 15–25% of diabetes patients develop diabetic foot ulcer (DFU) in their lifetimes, among which approximately 85% will undergo amputation [3,4]. DFU is a type of non-healing chronic wound resulting from the interplay of several factors either directly or indirectly caused by the hyperglycaemic condition. The abnormally high sugar level in the blood can result in poor blood circulation, prolonged inflammation, signalling factors irregularities, and high susceptibility to infection [5]. Altogether, these factors not only disrupt the normal wound healing phases but also form a feedback cycle that will eventually worsen the existing condition.

A DFU is a complicated wound that poses a challenge to conventional wound dressings, whereby it demands advanced therapies to address the specific requirements for wound treatment management. With the cost of treating ulcers involving infections and amputations being up to USD 45,000, a breakthrough in DFU management could have a significant impact on the overall healthcare budget [6]. Conventionally, the wound is managed with debridement of necrotic tissue and contaminants from the wound bed [7]. Then, a physical dressing is put in place to occlude the wound from drying out while preventing further contamination to the wound bed. However, debridement does not affect surface bacterial counts and it is also not ideal as a treatment [8]. Ultimately, both wound debridement and wound dressing aim to create an optimal environment for the wound to heal.

On top of wound debridement and dressing, the current gold standard for DFU treatment is a total contact-cast (TCC), whereby the leg is immobilised with a non-removable cast [9]. This will unload the pressure on the foot’s surface and prevents physical shear and stress that is harmful to the DFU patient. It usually takes an average of twelve weeks for complete wound healing using a TCC [10] or more depending on the severity of the DFU [11]. However, the disadvantages of using a TCC are that it limits the patient’s movement and it also requires special resources and trained caretakers for specific handling. Besides that, the wound is still highly susceptible to infection and this will delay wound healing after prolonged wear [9]. Currently, research has been performed to strategize and to find other potential alternatives to TCC [12] such as improved or enhanced wound dressings [13].

Although they are not the primary factors of chronic wound healing in DFU, a persistent infection and biofilm formation are commonly found in many clinical presentations [14]. The prevalence of biofilms in chronic wounds according to recent meta-analysis has been reported to be 78.2%, with some cases recording a higher rate of infection [15,16]. Bacterial infection triggers inflammation at the injury site following the recognition of bacterial antigen or secreted products, such as proteolytic enzymes [17,18]. The abnormal level of infection rate contributes to the delay in wound healing by prolonging the inflammatory phase as the immune cells attempt to clear the infection [19].

Infection management is typically the priority of DFU treatment. The antibiotics given to treat the ulcer bed should be based on the infected organism’s spectrum [20]. The typical infections found in DFU are *Staphylococcus aureus* and *Escherichia coli*, which can be eradicated by the administration of aminopenicillin and penicillinase inhibitor, along with clindamycin, quinolone, or metronidazole [21]. Intravenous injections such as gentamicin, imipenem, vancomycin, lenozoid, teicoplanin, or rifampicin are also potentially effective [22,23]. In addition, silver nanoparticles and other hard metals such as arsenic, copper, and zinc have been proven to exhibit antimicrobial properties. However, metal toxicity may be the main concern and potentially poses a risk [24].

Antibacterial medication can be administered post-debridement topically in the form of antiseptic cream, gel, or wound dressing or through systemic antibiotics to manage wounds with persistent infection [25]. In the context of DFU, which poses a high risk of prolonged infection, a new dose of antibacterial medication must be administered when the effect of the medication wears off. Hence, a delivery method that sustain antibiotic release to the wound area is preferable for DFU management. Furthermore, the diagnosis and management of DFU vary substantially according to the experience of the clinician. Many wound dressings have been designed to address DFU depending on clinical presentation [19]. Apart from covering the wound, many of the dressings interact with the wound environment to facilitate healing. In general, wound dressing for DFU should be able to address poor blood circulation, prolonged inflammation, irregularities of the signalling factors, and high susceptibility to infection issues [26].

Therefore, the efficacy of wound healing with interactive wound dressings relies heavily on the geometry and three-dimensional (3D) architecture of their microstructure. These dressings can be fabricated into various functional forms, including films, foams, hydrogels, and hydrocolloids. Each design interacts in its unique way with the wound environment. For example, a wound that has high exudation requires a high absorption ability of the dressing, such as in foam dressings. In contrast, a wound that has low exudation requires the moisture retention ability of a hydrogel dressing [27].

In recent years, the physical interactions between the wound dressing and microenvironment have been scientifically complemented with the biological properties from its origin. Natural-based biomaterials including collagen, hyaluronic acid, chitosan, alginate, and elastin in wound dressings caused a less immunogenic reaction and less toxicity, and a tissue-stimulating effect with optimum biodegradable properties [28,29,30]. One of the biomaterials that have been developed and widely used to treat DFU clinically is collagen. Collagen dressings can act as skin substitutes for the native extracellular matrix (ECM) to guide the complex cellular interaction necessary to prompt keratinocyte and fibroblast migration [31]. Collagen is the most abundant protein in human tissue, primarily in the skin and bone [32,33]. The collagen is readily harvested from marine, bovine, porcine, ovine, and equine sources [34], which can be easily fabricated into bioscaffolds of any design and 3D geometries [34,35]. The collagen biomatrix mimics the native collagen in ECM and stabilises the vascular and cellular components in the wound by reducing matrix metalloproteinases (MMP) levels that are typically imbalanced in chronic wounds while providing structural support for tissue repair [36]. In a previous study, Fauzi and co-workers, in 2016, demonstrated the ability of ovine collagen to be fabricated into film, foam, or hydrogel with favourable properties that enhance human dermal fibroblast and epidermal keratinocyte attachment, and they are well-distributed on various designs of 3D bioscaffolds [37,38,39,40]. The mechanisms in which the collagen-based dressing improves wound healing include the abilities to bind to the growth factors, regulate activities of the cells, facilitate communication intracellularly, and serve as a physical structure to aid tissue repair in acute and chronic wounds [41]. The collagen bioscaffolds were revealed to be biocompatible, biodegradable, and non-cytotoxic with adequate tensile strength to support positive outcomes in wound healing treatment in rodents [42].

Various collagen-based dressings have been developed with the main aim of continually improving their effectiveness. One of the examples is to integrate antibacterial functionality to reduce infections that are known to exacerbate chronic wounds. A collagen-based dressing impregnated with antimicrobial agents addresses the high exudation with a high risk of bacterial infection in DFU [43,44,45,46]. The current study sought to verify the wound healing potential of an antibacterial-impregnated collagen sponge with a porous 3D microstructure for the treatment of DFU via a systematic review of previous literature.

## 2. Materials and Methods

This systematic review was carried out using the Preferred Reporting Items for Systematic Review and Meta-analyses (PRISMA) [47] adopted with some modifications from Holmes and co-researchers (2013) [36].

### 2.1. Search Strategy

A systematic review of the literature was conducted to identify relevant studies about the reported effect of porous collagen-based dressing fortified with the antibacterial or antimicrobial agents on wound healing for DFU patients. Four databases, namely, Scopus (Elsevier, The Netherlands), PubMed (National Center for Biotechnology Information (NCBI), Bethesda, MD, USA), Ovid MEDLINE(R) (National Library of Medicine (NLM), Bethesda, MD, USA), and ISI Web of Science (Clarivate Analytics, Philadelphia, PA, USA), were used to search for relevant articles within the last ten years (from 2010 to 2020). In all databases, a combination of controlled terms from MeSH (Medical Subject Headings) terms was defined using the PICO strategy whereby Population (P) was diabetic foot ulcer, Intervention (I) was porous collagen-based dressing fortified with the antibacterial or antimicrobial agent, and Outcome (O) was wound healing. The Comparison (C) item was agents.

The search strategy used four sets of keywords, namely, (1) collagen and (2) foam or sponge or matrix or por*s and (3) antib* (to obtain antibiotics or antibacterial records) or antimic* (to obtain antimicrobial records) and (4) diabetic or foot or ulcer or lower extremity or chronic or wound. The comprehensive search strategy is displayed in Table 1 (based in Ovid MEDLINE and adapted to the other databases). The search was conducted three times to ensure all relevant papers were able to be identified.

### 2.2. Inclusion Criteria

The inclusion criteria mandated original research articles: clinical studies including randomised controlled trials and observational studies. The selected studies had to provide evidence on (1) the use of antibacterial collagen-containing or collagen-based porous dressing, and (2) have wound healing outcomes among (3) the diabetic foot ulcer population. There was no restriction on the aetiology of the diabetic ulcer. The articles also had to be written in English, contain abstracts, and have been published within the past 10 years (year 2010–July 2020) based on the search settings.

### 2.3. Exclusion Criteria

Review articles, editorials, news, conference papers, case reports, and letters were excluded. Articles that did not meet the inclusion criteria were also excluded.

### 2.4. Study Selection

Papers were screened in three phases to complete a part of this systematic review. Firstly, after removing duplicates and review articles, the initial screen was performed by omitting studies based on titles that did not match the inclusion criteria. The second phase included screening the abstracts of the remaining papers for unmatched inclusion criteria. The final phase was excluding any papers that did not meet the inclusion criteria after full-text reading by two independent reviewers (I.N.A. and A.N.). The initial step was title and abstract screening, and was followed by the final step of full-text reading of the selected articles. Any irrelevant studies were excluded due to no data presented on diabetic ulcers or the porous-like structure of a collagen-based material with the addition of an antibacterial ingredient. The disagreements between reviewers were resolved by a detailed discussion.

### 2.5. Data Extraction and Management

Data collection was standardised using a data extraction table. Data recorded from the studies were as follows: (1) study aim, (2) study design, (3) product description, (4) sample size with a sample of diabetic ulcer patients included in the analysis, (5) time of measurement and duration of the study, (6) a brief description of the study results, and (7) a brief conclusion of the study. Some studies included other aetiologies for chronic wounds, from which only data for DFU were extracted.

### 2.6. Risk of Bias Assessment

A quality of method assessment to check for risk of bias was scrutinised using the Scottish Intercollegiate Guideline Networks (SIGN) (Healthcare Improvement Scotland (HIS), Glasgow, Scotland, UK) randomised control trial and cohort checklist [48]. The method was adopted from a previous systematic review by de Oliveira and co-researchers (2018) [49]. The assessment includes the methodology criteria of (1) a clinical trial, (2) patient selection, (3) outcomes, including evidence of statistical analysis, and (4) integrity criteria, such as institutional ethical approval, disclosure of conflicts of interest, and funding.

## 3. Results

### 3.1. Search Results

**Figure 1:**
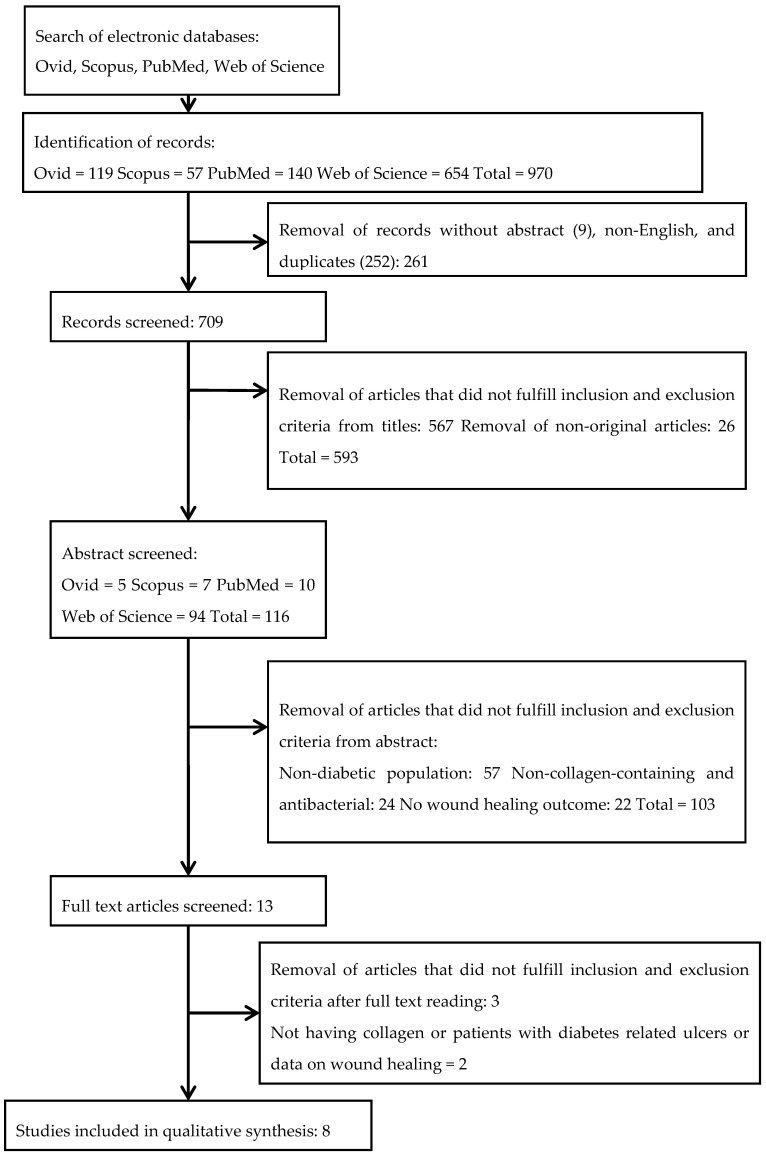
Flow chart of the screening process.

Initially, a total of 970 articles were identified as potentially relevant articles. A total of 252 duplicate articles and an additional 26 review articles were removed. Two reviewers independently assessed all articles for inclusion or exclusion criteria based on the titles and abstracts. In total, 593 articles were removed based on titles because the studies were not original articles or did not pass the inclusion and exclusion criteria. An additional 103 were removed after reading the abstracts: they did not (1) address treating a diabetic population, (2) describe antibacterial and collagen-containing porous dressings, or (3) address wound healing outcomes according to the inclusion and exclusion criteria. After reading the full texts, five articles were excluded for not fulfilling the inclusion criteria. One article was identified as a previous version [50] of a current study by Uckay and co-workers (2018) [51,52]. Finally, eight articles were eligible to be reviewed. A flowchart of the screening process, with the exclusion reason(s), is presented in Figure 1.

### 3.2. Study Characteristics

The summary of all studies’ characteristics involved is displayed in a data extraction table in Table 2. All studies were published between 2012 and 2019. Five types of antimicrobial collagen-based dressings were identified in the eight articles selected: (1) (*n* = 3) gentamicin-loaded collagen dressing (Garamycin, Innocoll Pharmaceuticals Ltd. and Collatamp^®^EG, Syntacoll, Germany) [51,52,53]; (2) (*n* = 2) collagen dressing impregnated with antimicrobial polyhexamethylene biguanide (PHMB) (PuraPly AM, Organogenesis Inc., Canton, MA, USA) [54,55]; (3) (*n* = 1) collagen with mixture of cellulose and silver (Promogran Prisma, Systagenix Wound Management Ltd., Skipton, UK) [56]; (4) (*n* = 1) mixed collagen with gentian violet/methylene blue (Hydrofera Blue, Hollister Wound Care Inc, Libertyville, IL, USA) [57]; (5) (*n* = 1) bovine-derived collagen impregnated with silver ions (Ag) (Puracol plus Ag, Medline Industries, Northfield, IL, USA) [58].

### 3.3. Gentamicin on Collagen Dressing

Gentamicin is an antibiotic from the aminoglycoside class that is active against many strains of Gram-positive and Gram-negative pathogens [59]. As an aminoglycoside, gentamicin has shown a rapid bactericidal and post-antibiotic effect, inoculum-independent activity, synergy with beta-lactam and glycopeptide antibiotics, and easy dosing [60]. With respect to collagen-based dressings with antibiotics, gentamicin is the most frequently investigated antibiotic and is clinically approved worldwide [61]. Gentamicin-containing collagen sponges have been previously shown to be effective at preventing infections in clinical cases [60,62]. The common biomechanism of the collagen sponge’s application is to be gradually degraded by native collagenase and absorbed systemically. Besides that, gentamicin has been proven to be initially released through passive diffusion and later actively by the biodegradation of the sponge collagen [53].

The effectiveness of topical gentamicin-collagen sponge was analysed in two studies from the same authors with different parameters: (1) mildly infected DFU intervention with only the sponge [52], and (2) moderate or severely infected DFU intervention including therapy with systemic antibiotics [51]. The patients all had diabetic ulcers with varying degrees of infection. Uckay’s previous work on moderate or severe infection is an updated and expanded version of Lipsky and co-researchers (2012) [50]. Both studies combined the use of a gentamicin collagen sponge with various systemic antibiotic interventions for a synergistic effect. The articles also included assessing the safety and cure rates for more than a month instead of the previously reported study for just a week. Both of Uckay’s findings displayed no significant difference in the treatment group compared to the control towards the overall cure. In contrast, their previous study concluded that the treatment had a higher probability for a clinical curing and pathogen eradication of infected DFU. However, Varga et al. [53] using another gentamicin collagen sponge product in conjunction with systemic antibiotic treatment reported a significant reduction of wound recovery of almost two weeks in their patients compared to almost five weeks in the nontreated group. However, patients in Varga’s study were not treated on non-healing DFUs, but rather recent and minor amputated regions of the ulcer, which may have altered conditions of the wound bed.

Briefly, in a pilot study considering mild DFU, Uckay and co-researchers (2018) [51] proved no significant difference between the treatment using a gentamicin-collagen sponge (*n* = 11) and no treatment as a control (*n* = 11) for mild DFUs under a pilot study of 22 patients. The investigation did not involve any systemic antibiotic, instead emphasizing solely on the efficacy of using a topical gentamicin collagen-based dressing [52]. The study demonstrated 91% (*n* = 20) and 9% (*n* = 2) of 22 patients improved and did not improve, respectively. However, 56% of the total patients successfully had the pathogen eradicated in 14 days (depending on appointments for over 24 days). Both the sponge and control arms resulted in 91% wound closure each with no difference in pathogen eradication rate. However, the wound duration and size of each patients were not reported. In support, Varga’s study also reported no significant differences in microbial findings between their treatment and control arms.

The three most frequently isolated pathogens from the wound were *Staphylococcus aureus* (*n* = 8), *Pseudomonas aeruginosa* (*n* = 4), and *Staphylococcus epidermidis* (*n* = 3), with eight patients (36%) showing polymicrobial infection. All patients were not feverish and there was a slight reduction of median leukocyte count from 9.9 to 7.8 G/L. Instead of the wound area, a customised wound score was used to include inflammatory parameters such as wound size, duration, pus, and pain. There is an overall trend of decreased wound score trend during the study from 13 to 7 points indicating wound healing; however, no statistically significant difference was achieved. Similarly, Varga et al. reported the most cultivated microbials were *Staphylococcus aureus* (*n* = 14), *Enterococcus faecalis* (*n* = 6), *Klebsiella* spp. (*n* = 5), and *Pseudomonas aeruginosa* (*n* = 4), and there were no adverse effects of the gentamicin and systemic antibiotic administration.

In another study, Uckay et al. (2018) [52] included both moderate (*n* = 77) and severe (*n* = 11) DFU patients but reported no significant difference between both groups. At the end of the randomised clinical trial, 73% of the total 88 patients achieved a clinical cure presentation. Among the treatment (*n* = 43) and control (*n* = 45) groups, 15% showed improvement; however, only 52% had total pathogen eradication after being treated weekly between 14–28 days. Besides that, 8% were stagnated and 5% had worsening wounds due to ischemia. The systemic antibiotics levofloxacin (*n* = 48), amoxicillin (*n* = 58), linezolid (*n* = 6), metronidazole (*n* = 6), and clindamycin (*n* = 3) were used; however, the types of patients receiving the antibiotics were not specified. However, for other severe infections, such as sepsis, the piperacillin tazobactam (*n* = 3) was used.

The four most frequently isolated pathogens from the wounds were *Staphylococcus aureus* (*n* = 41), *Pseudomonas aeruginosa* (*n* = 5), *Escherichia coli* (*n* = 9), and streptococci (*n* = 11) in this study; 37 patients (42%) presented with polymicrobial infections. The infected DFU patients were treated with systemic antibiotics for a median of 21 days. There was no significant difference in the outcomes observed for treatment with (88%) or without (87%) with a gentamicin-sponge, nor was there one in the rates of pathogen eradication for the two groups. The wound score decreased from 18 to 8 points but was not significant. However, a trend toward rapid wound healing was reported when using a gentamicin-collagen sponge in weeks 3–5 compared to no use of the sponge. Some of the patients (*n* = 10) had pressure off-loading therapy; this therapy could have significantly affected the results. Furthermore, out of the 88 patients, 20 had minor adverse events such as gout (*n* = 1), nosocomial pneumonia (*n* = 1), and worsening arterial insufficiency (*n* = 5), and thus could not be included in the final analysis.

### 3.4. Collagen with Polyhexamethylene Biguanide

Polyhexamethylene biguanide hydrochloride (PHMB) is a cationic antimicrobial against both Gram-positive (*Staphylococcus aureus*) and Gram-negative (*Pseudomonas aeruginosa*, *Escherichia coli*) bacteria. Thus, PHBM is able to bind strongly to the bacterial cell walls and membranes by disrupting their transport, biosynthesis, and catabolic functions [63]. It can also bind to the biofilm matrix components and increases its concentration during application by providing a toxic environment to the bacteria [64]. However, PHBM demonstrated minimum toxicity when applied to the skin or wounds [65,66] and was previously proven to be effective to wounds of various aetiologies [67]. This outcome is supported by other two prospective clinical studies performed by Bain and the team (2019) [54] and Oropallo (2019) [55], whereby both studies used porcine collagen matrix with PHMB to intervene with the chronic wounds of various aetiologies.

The prospective study by Bain and co-researchers (2019) [54] used 63 patients with venous ulcer (*n* = 18), both trauma and laceration (*n* = 14), postsurgical wound (*n* = 10), pressure injury (*n* = 8), chronic vascular wound (*n* = 2), diabetic ulcer (*n* = 6), and other categories (*n* = 2). Focusing on those with DFU, 66.7% of the six DFU patients achieved complete wound closure by week 12. The complete wound closure for all types of wounds was five weeks for wounds that were at a minimum of four months in duration. No hypersensitivity issues were presented, and the antimicrobial component was biocompatible with no signs of infection. Post-market evaluation with a positive influence on healthcare costs was also noted.

These findings were similar to those summarised by Oropallo (2019) [55] whereby out of 41 patients, 18 patients had pressure ulcers, nine patients had surgical wounds, five patients had venous leg ulcers, four patients had DFU, and five patients presented with other wound types; 25% of the four diabetic patients achieved complete wound closure at week 12, as reported by Bain et al. [54]. The mean wound closure was 43% at week 4 and 50% at week 12. Meanwhile, for other types of wounds, 73% wound area reduction and 37% fully complete wound closure were reported. Thus, the current scenario took an average of 6.7 weeks for non-healing wounds on average of 24 months. However, both studies did not have a comparable control group and the statistical significance was not described.

### 3.5. Hybrid Collagen-Cellulose Integrated with Silver

Collagen with oxidised regenerated cellulose (ORC) has previously been shown to restore the wound microenvironment and its biochemical imbalances [68]. The mechanism is thought to work through binding with the inactivating proteases, including MMPs and elastase [69,70]. This scenario is critical for chronic wound healing, such as DFU. Additionally, silver has been extensively studied as an antimicrobial agent to control infection and is increasingly gaining interest in biomedical applications because it does not contribute primarily to bacterial resistance, as speculated in antibiotics [71].

A study by Gottrup et al. (2013) [56] included 39 patients to test for the effectiveness of hybrid ORC and silver. The study output showed a distinctive significant difference, whereby 79% of the treatment group (*n* = 24) achieved more than half of wound closure compared to 43% in the control group (*n* = 15) by week 4 in the 14-week study. However, three patients could not complete the study because of certain factors, including death, nurse strike, and illness, and that could have skewed the results. In addition, by the end of the study, for wound size reduction of more than 50%, the treatment group (91%) showed a larger percentage of healed wounds compared to the control group (69%) (*p* < 0.05). For complete wound closure, the treatment group (52%) achieved a higher percentage than the control group (31%) by week 14. The control group had four further withdrawals from infection, while no withdrawal was observed in the treatment group. Those with less than 50% wound closure had higher elastase (*p* = 0.0295) and MMP-9 (*p* = 0.028) by week 4, but no difference in MMP-9-TIMP-1 (metallopeptidase inhibitor-1). This indicates that the collagen matrix stabilises the extracellular matrix proteases and that non-healed wounds have higher concentrations of MMPs and elastases [72]. No adverse effects were found in this study after using the product.

### 3.6. Collagen Dressing Gentian with Violet/Methylene Blue

Gentian violet/ Methylene Blue (GV/MB) are dyes with antibacterial, antifungal, and immune therapeutic characteristics while using a dressing to primarily absorb wound exudate and protect the wound from external microorganisms. The GV/MB antibacterial polyurethane was applied over an ovine-based collagen matrix dressing as a temporary extracellular matrix support [73]. In a previous study, Lullove (2017) [57] studied 53 patients with various chronic lower extremity wounds that failed to heal for at least four weeks. Out of 53 patients, 22 patients had diabetic ulcers, 29 patients had venous leg ulcers, and 3 patients had pressure ulcers. For DFU patients, half of the patients had achieved more than 40% wound area reduction at week 4. By week 12, 40.9% of patients had achieved more than 80% wound area reduction. However, only 59.1% of the patients achieved complete wound closure by week 12; 95.5% of the patients achieved complete wound closure by week 20; and the last patient healed completely after week 24. All patients eventually achieved complete closure over a maximum of 24 weeks, with the average time to closure being 10.6 weeks. No adverse reactions were reported. The bacterial load showed a trend for increment except for one case, but there was no significant difference and it was only between two limited endpoints of week 0 and week 4.

### 3.7. Collagen Integrated with Silver

The prospective clinical study by Manizate and co-researchers [58] compared the efficiency to reduce bacterial load between two products of sodium carboxymethylcellulose with 1.2% ionic silver (CMC) and bovine native collagen with 1.12% ionic silver (BDC). Silver (Ag) ions are known to be bactericidal by binding to the bacterial cell wall, respiratory and nutrition-involved proteins, and DNA—preventing the replication system [74]. Both dressings are meant to be able to modulate the wound bed. CMC plus Ag retains moisture and prevents fibrin ingrowth while giving adhesion, and BDC promotes healing through balancing the MMP levels [58]. Out of 10 patients, nine had bilateral venous stasis ulcers and only one had bilateral diabetic ulcers. Results reported that 50% of the wounds had *Staphylococcus aureus*; however, the bacterial rates did not impact the wound closure rate, and there was no significant difference in the bacterial amount over four weeks. The rate of wound closure for BDC was slower than CMC’s: 10.2% and 11.7% per week, respectively. There was no significant difference in the total percentage of wound closure between the two dressings. However, BDC was preferable and superior based on patient surveys, with 80% of patients describing lesser pain levels compared to CMC. There were no significant differences between the dressings for other measures, including removal ability, level of exudate, surrounding skin condition, and erythema or maceration. However, this study reported the continuation of using BDC compared to CMC in their clinical practice and was also requested by their patients. The selection was based on higher granulation tissue formation, newly formed tissue, and convenience observed (no presented measurement).

### 3.8. Quality of Articles Methodology

The quality of methodology was scored based on the established guidelines and the assessment table is displayed in Table 3. The great majority of the selected studies fulfilled more than 50% of established standards. Only one study reached 50% of the standards. The randomised trials had superior results in terms of minimising risk, by achieving more than 70% of the established standards. Notably, each article was funded by a respective company from which the products were provided.

## 4. Discussion

The findings from this evidence-based review reported a trend of healing rate incrementation using collagen dressing loaded with antimicrobial components for DFU. However, the antimicrobial component may not significantly contribute to the positive effects displayed, as no difference in terms of pathogen eradication was observed in four studies due to the lack of antibacterial assays in these reports. Only one study demonstrated reduced bacterial infection with the silver-loaded collagen sponge. Moreover, other studies did not measure the level of infection pre- and post-intervention with their hybrid collagen sponge. Hence, it is difficult to link reports on the enhancement of wound healing using antimicrobial-loaded collagen sponges with the antimicrobial properties.

Nevertheless, none of the dressings presented with initiation or worsening of bacterial infection on the DFU wound, implying the efficacy of the antimicrobial agent to prevent infection. In addition, Bain and co-workers (2019) did not detect any hypersensitivity issue with the polyhexamethylene biguanide-loaded collagen sponge, suggesting the safety of antimicrobial-loaded collagen sponge for DFU application [54]. Unfortunately, none of the other studies measured this outcome, making it impossible to determine the suitability of other antimicrobial-loaded collagen sponges in DFU use.

The clinical data are limited by small sample size and poor design factors, such as the lack of a standard healing time, a standard wound, a standard treatment duration, and an appropriate control group; variation in outcome measures; variation in the sizes of the wound; and variation in the standard of wound care. For example, both Bain and co-researchers (2019) and Oropallo (2019) included patients with various comorbidities present, such as hypertension, diabetes mellitus type 2, hyperlipidemia, peripheral vascular disease, coronary artery disease, and chronic kidney disease [54,55]. Besides, 22 patients in Oropallo’s study had diabetes mellitus type 2, but only four had diabetic-related foot ulcers, which could have contributed to selection bias. Bain and research team (2019) also did not have a protocol to specify how the investigator should perform normal wound care using the porcine collagen matrix with PHMB, thereby leaving room to considerable variability. Some patients received adjunct therapies such as systemic antibiotics or hyperbaric oxygen, which may have affected wound healing results. For example, Uckay and co-workers (2018) [51] and Varga et al. (2014) [53] had patients take various systemic antibiotics but did not standardise the therapies among the different patients. In addition, the studies’ outcome measurements were customized based on the wound scores and were also not standardised to other clinical trials. Additionally, there was a lack of standardisation in patient selection. Excluding specific significant comorbidities may improve study outcomes but also diminish the potential to improve wound care products. Many of the limitations in the clinical trials for wound healing products are supported by another systematic review [75].

It is difficult to make conclusive statements on the efficiency of antimicrobial-integrated collagen bioscaffolds specifically to treat DFU in terms of wound healing and the reduction of bioburden and proteases in the MMPs. There is no evidence that these products should replace the gold standard of TCC, although they may be more cost-effective compared to other diabetic wound management strategies, such as aetiology identification, infection management, adequate vascular supply, regular debridement, and offloading [76]. In terms of cost, Lullove (2017) [57] calculated the average cost for a single wound to be close to $2749.49, considerably less than a previous study using the US Wound Registry, which reported that the mean cost of unhealed wounds was estimated to be $4000 for each patient at 6 months, and increased to $18,000 after 2 years of wound duration. [77].

The ideal wound dressing should ensure the appropriate bactericidal effect, wound moisture, and maintenance of extracellular matrix levels; protect wound beds from excessive slough; be readily available; and be easy to apply and adhere. There is not enough evidence to prove the superiority of one dressing, antimicrobial component, collagen source, or combination over another. The current studies fail to identify the necessity for combining products with offloading to achieve healing in a standardised manner. The use of these dressings for the specific severity and levels of infection of diabetic ulcers also failed to be conclusive. Future work should consider the inclusion of bioburden levels, biofilm activity, and enhancement of extracellular levels. Finally, for wound care to be streamlined in management, specific levels of patient severity and aetiologies or the most vulnerable patient populations could be targeted to better reflect current practice.

## 5. Conclusions

The evidence generally shows positive outcomes for using a collagen-based dressing integrated with antibacterial components, whereby more DFU wounds were healed compared to without dressing. However, the level of efficiency for treating DFUs with varying severity and different infection levels requires stronger evidence.

## Figures and Tables

**Table 1:** Search strategy based on Ovid MEDLINE for systematic literature search.

No	Keywords
1	collagen
2	foam
3	sponge
4	matrix
5	por*s
6	antib*
7	antimic*
8	diabetic
9	foot
10	ulcer
11	lower extremity
12	chronic
13	wound
14	Or/2–5
15	Or/6–7
16	Or/8–13
17	1 and 14 and 15 and 16
18	Limit 17 to yr. = “2010–2020”

**Table 2:** A summary of studies of antibacterial sponge-like scaffolds for chronic wound healing.

Study	Aim	Product	Study Design	Total Sample Population (DFU)	Time	Results	Conclusion
Bain et al. 2019 [54]	Native type I Collagen with Polyhexamethylene Biguanide (PHMB) antimicrobial	PuraPly AM, Organogenesis	Prospective, Registry, post-market clinical evaluation	63 (6)	Weekly for 24 weeks	66.7% diabetic patients showed complete closure by week 8 No hypersensitivity issues, antimicrobial component biocompatible Positive influence on healthcare costs	Porcine collagen matrix with PHMB (PCMP) positively impact wound healing various types of lesions, including DU as adjunct therapy
Oropallo, AR. 2019 [55]	Native type I Collagen Matrix with Polyhexamethylene Biguanide (PCMP) ability to close DFU wounds over 12 weeks.	PuraPly AM, Organogenesis	Prospective	41 (4)	Weekly (if necessary) for 12 weeks	25% of DFU achieved complete closure. The mean wound closure for DFU were 43% at week 4 and 50% at week 12.	PCMP treatment responds positively to nonhealing chronic wounds including diabetic ulcers.
Uckay et al. 2018 [52]	Patients with mild-DFU treated with topical gentamicin and collagen sponge	Garamycin, Innocoll Pharmaceuticals Ltd.)	Randomized controlled trial	24 (22)	14 different days over 24 days	91% cured clinically 56% had pathogen eradication at end of study Most frequent isolated pathogens were *S. aureus, P. aeruginosa,* and *S. epidermis.* 36% patients had polymicrobial infection.	There was no difference between the treatment and control for treating mild diabetic ulcers, but it was well tolerated.
Uckay et al. 2018 (1) [51]	Patients with moderate or severe DFU infection treated with topical gentamicin and collagen sponge and systemic antibiotic therapy	Garamycin, Innocoll Pharmaceuticals Ltd.	Randomized controlled trial	88 (68)	Weekly for 14–28 days	73% were cured clinically 15% improved significantly 52% had total pathogen eradication	There was no significance in the overall cure compared to control although the sponge was well tolerated.
Gottrup et al. 2013 [56]	Collagen/oxidized regenerated cellulose (ORC)/silver treatment compared to standard	Promogran Prisma, Systagenix	Randomized controlled trial	39 (36)	Every 2 weeks for 14 weeks	79% had ≥50% reduction in wound area by week 4 compared to standard (43%) Levels of elastase decreased significantly MMP-9 reduced Lower MMP-9-TIMP-1 trend Improved healing rates	Collagen/ORC/silver increased healing rates and decreased levels of infection significantly.
Lullove, EJ. 2017 [57]	To improve chornic wounds in the lower extremity using ovine-based collagen matrix with gentian violet/methylene blue antibacterial dressings	Hydrofera Blue, Hollister	Retrospective case series	53 (22)	Twice weekly for 4 weeks then weekly until closure 24 weeks	76.5% average wound closure by 8 weeks 50% DFU patients had more than 40% wound closure in 4 weeks Average 10.6 weeks to closure 100% Re-epithelisation of all wounds at week 20 except 1 case achieved at week 24. Absence of re-infection on wounds Average cost for single wound to closure is $2749.49	Using collagen-based matrix with antibacterial foam healed more than 40% of chronic wounds within 4 weeks with absence of infection and adverse effects.
Manizate et al. 2012 [58]	Treat infected DFU with bovine-derived collagen and ionic silver (Ag) dressing hybrid (BDC) compared to carboxymethylcellulose and Ag dressing (CMC)	Aquacel Ag, Medline	Prospective; post-market clinical evaluation	10 (1)	Changed daily and checked at week 1 and 4 for 8 weeks	The absolute wound closure rate of BDC were higher than CMC but percentage of closed wounds were not significant *S aureus* accounts for 50% of wounds with most frequency Initial bacterial load did not have any significant effect on the closure rate	There were no statistically significant differences in terms of efficacy and effect of bioburden between the two dressings, but both showed positive trend for wound healing, with BDC more preferred by patients and more superior absolute wound closure.
Varga et al. 2014 [53]	Treat minor amputated diabetic patients with gentamicin collagen sponge	Collatamp^®^EG; Syntacoll	Prospective, randomized trial	50 (22)	Regular follow up with patients until wounds fully heal up to 20 weeks	Using the gentamicin collagen sponge shortened wound healing duration significantly by close to 2 weeks compared to those without the treatment General reduction of microbial findings in treatment group but no significant differences of bacterial loading between treated and non-treated group.	Although wound healing improved significantly with the gentamicin collagen sponge, there were no improvements to shortening the length of hospital stay, revision for wound breakdown or re-amputations.

Legend: DFU = diabetic foot ulcer; MMP = matrix metalloproteinases; TIMP = protease inhibitor.

**Table 3:** Bias criteria of methodology.

Study	Bain et al. [54]	Oropallo [55]	Uckay et al. [52]	Uckay et al. (1) [51]	Gottrup et al. [56]	Lullove [57]	Manizate et al. [58]	Varga et al. [53]
*Clinical Trials (Randomized and Cohort) Methodology*								
Does the study have a focused question?	Y	Y	Y	Y	Y	Y	Y	Y
Is the study randomized with appropriate concealment method?	X	X	Y	Y	Y	X	X	Y
Was there some recognition that assessment of outcome could have been influenced by exposure status when blinding was not possible?	X	Y	Y	Y	Y	X	Y	X
Groups being studied (same or different sites) are selected from comparable source populations in all respects other than the investigated factor?	Y	X	Y	Y	Y	X	X	Y
Was blinding about treatment allocation performed for subjects and investigators?	X	X	Y	Y	Y	X	X	X
Was the only difference found is between groups the investigated treatment, i.e., large variables	X	X	Y	X	Y	X	X	X
How many percentage (Not more than 20%) of participants or clusters in each arm of study dropped out before study completion?	7.9%	0%	8.3%	23%	7.7%	0%	0%	10%
Was there a comparison made between participants throughout the entire study and those that were lost to follow up or dropped out (by exposure status)?	Y	N/A	N/A	Y	X	N/A	N/A	N/A
Other sources was used as evidence to demonstrate valid and reliable method of outcome assessment.	Y	Y	Y	Y	Y	X	X	Y
All relevant outcomes were clearly defined, are measured in a reliable, standard, and valid way	Y	Y	Y	Y	Y	Y	Y	Y
How well was the study done to minimise bias?	X	X	Y	Y	Y	X	X	X
*Clinical Trials Selection of Subjects*								
Does the study have inclusion/exclusion criteria?	Y	Y	Y	Y	Y	Y	Y	Y
Number of samples	Y	Y	Y	Y	Y	Y	Y	Y
Age (mean or median)?	76	62	70	71	60	75.9	X	62
Sex of DU patients?	?	?	Y	Y	Y	?	X	Y
Control or comparison with another treatment or standard of care?	X	X	Y	Y	Y	X	Y	Y
Was there a baseline wound condition for selection?	Y	Y	Y	Y	Y	Y	Y	Y
Type of diabetes	X	Y	X	Y	X	X	X	Y
Was ankle brachial index measured and reported before selection?	X	X	Y	Y	Y	Y	Y	X
Was the HbA1c measured and reported?	X	X	Y	Y	Y	X	Y	Y
What was the median baseline wound area?	6.5 cm^2^	7.2 cm^2^	Y	Y	4.3 cm^2^	6.4	14.9	X
Minimum wound duration?	4 months	24 months	X	X	1 month	1 month	X	N/A
*Outcomes Criteria*								
Wound closure analysis?	Y	Y	?	?	Y	Y	Y	?
Time to complete wound closure were measured appropriately?	Y	Y	X	X	X	Y	Y	Y
Did the study report data on either tissue granulation or exudates?	X	X	?	?	Y	X	X	X
Were there any results pertaining microbiological, pathogen, or biofilm data?	X	X	Y	Y	X	X	Y	Y
Does the study include an inferential statistical analysis?	X	Y	Y	Y	Y	X	Y	Y
*Other Integrity Criteria*								
Was there Institutional Ethical consideration?	Y	Y	Y	Y	Y	Y	Y	Y
Where there mentions of funding?	?	Y	Y	Y	Y	Y	Y	X
Location of Site (clinical) mentioned	X	Y	Y	Y	Y	Y	X	Y
Year of data collection (clinical)	X	N	Y	Y	Y	Y	X	Y
Did authors declare/disclosure conflict of interest?	Y	Y	Y	Y	Y	Y	Y	X
*Ratio*	16/32 (50%)	19/32 (59%)	25/32 (78%)	27/32 (84%)	29/32 (91%)	17/32 (53%)	18/32 (56%)	19/32 (59%)

Legend: (X) inadequate; (Y) adequate; (?) unclear; and (N/A) not applicable.
